# Hypoglycemic and hypolipidemic activity of aqueous leaf extract of *Passiflora suberosa* L

**DOI:** 10.7717/peerj.4389

**Published:** 2018-02-20

**Authors:** Hasani Prabodha Sudasinghe, Dinithi C. Peiris

**Affiliations:** Department of Zoology (Centre for Plant materials & Herbal Products Research), University of Sri Jayewardenepura, Nugegoda, Western Province, Sri Lanka

**Keywords:** *Passiflora suberosa*, Aqueous leaf extract, Hypoglycemia, Hypercholesterolemia

## Abstract

Leaves of *Passiflora suberosa* L. (Family: Passifloraceae; common name: wild passion fruit, devil’s pumpkin) are used in Sri Lankan traditional medicine for treating diabetes. The present study investigated the *in vivo* ability of *P. suberosa* leaves to manage blood sugar status and associated cholesterol levels. Mechanisms of action and toxicity were also determined. Phytochemical screening of aqueous extracts of *P. suberosa* leaves and carbohydrate content of the leaves were determined according to previously published methods. In two group of male mice (*n* = 9), effects on fasting and random blood glucose levels (BGLs) of different acute doses (0, 25, 50, 100 and 200 mg/kg) of the aqueous leaf extract (ALE) were evaluated at 1, 3, and 5 h post-treatment. In another set of mice, the fasting BGL was evaluated following treatment of 0 or 50 mg/kg ALE (dose prescribed in traditional medicine) for 30 consecutive days. The lipid profile, some mechanism of ALE action (diaphragm glucose uptake, glycogen content in the liver and skeletal muscles) and its toxicity (behavioural observation, food and water intake, hepatoxicity) were also assessed following 30-day treatment. However, sucrose and glucose tolerance tests and intestinal glucose uptake were conducted to determine portion of mechanisms of action following single dose of 50 mg/kg ALE. Phytochemical screening revealed the presence of alkaloids, unsaturated sterols, triterpenes, saponins, flavonoids, tannins and proanthocyanidins. Carbohydrate content of the leaves was 12.97%. The maximum hypoglycemic effect was observed after 4 h of 50 and 100 mg/kg ALE administration. The extract decreased fasting BGL (18%) following an oral sucrose challenge and inhibited (79%) glucose absorption from the intestine. Correspondingly, the levels of glycogen in the liver (61%) and in the skeletal muscles (57%) were found be higher than that of the control group. The levels of total cholesterol (17%) and tri-glyceraldehyde levels (12%) found to be reduced in treated groups. Furthermore, no significant toxic effects were observed in treated groups. The present results suggest that the leaves of *P. suberosa* can be used to manage blood glucose and cholesterol levels. Isolation of active compounds are recommended for further analysis.

## Introduction

The term diabetes mellitus (DM) describes elevated blood glucose levels (hyperglycaemia) associated with a relative insulin deficiency, insulin resistance, or both (Ballinger, 2011). The global spread of DM as a chronic disease has contributed to its pandemic characteristics. Diabetes is a serious metabolic syndrome and also considered as a leading cause of mortality in the world. Globally, the prevalence of DM in 2016 was 8.5% of the adult population (422 million adults) and the numbers are projected rise up to 9.9% by the year 2045 (World Health Organization, 2017a; World Health Organization, 2017b). Diabetes is prevalent specially in South Asian countries like Sri Lanka, with one in five adults suffering from either diabetes or pre-diabetes (Katulanda et al., 2008). According to recent statistics, the prevalence of diabetes among adults in Sri Lanka 8.5% with one in 12 adults suffers from diabetes, which totals to 1.16 million (Bandara, 2016). High blood glucose levels are also associated with decreasing the levels of High-Density Lipoprotein cholesterol and escalation of Low-Density Lipoprotein cholesterol, thus increasing risk of coronary heart diseases. Therefore, it is vital to manage both diabetes and lipid levels. The treatment for DM consists of the administration of either insulin or other hypoglycaemic agents in conjunction with recommendations for dietary control and physical exercise. However, these hypoglycaemic agents can cause adverse effects such as hypoglycaemia, gastrointestinal disorders, renal toxicity and hepatotoxicity (Caprio & Fonseca, 2014). These limitations of synthetic agents have led to consideration of alternative therapies, including herbal formulations. Medicinal plants have become increasingly important in primary health care. In fact, the WHO estimated that 80% of the population living in the developing countries like Sri Lanka relies exclusively on herbal medicine for their primary health care (World Health Organization, 2017a; World Health Organization, 2017b). Medicinal plants have become popular because of their less side effects, for the presence of secondary metabolites, which may have numerous biological activities against numerous disorders, including infertility (Peiris, Dhanushka & Jayatilleka, 2015), cancer and various infectious diseases (Fodouop et al., 2015). Many pharmacological investigations are conducted to identify new drugs for the treatment of these diseases. Moreover, many of these plant species have largely been used in traditional medicinal systems over hundreds of years to treat DM.

Plant species of genus *Passiflora* (Family Passifloraceae) are known to contain many active components of therapeutic value and are widely used as biologically active agents against a number of diseases (Dhawan, Dhawan & Sharma, 2004). Leaves of *P. edulis* (Colomeu et al., 2014), *P. alata* Curtis (Figueriedo et al., 2016) and *P. incaranata* (Gupta et al., 2012a; Gupta et al., 2012b) and peels and seeds of *P. edulis* exhibit strong antidiabetic activities (Kandandapani, Balaraman & Ahamed, 2015). Passiflora *suberosa* is commonly known as wild passion fruit, devil’s pumpkin or indigo berry. *P. suberosa* is a climber with a gelatinous stem that becomes corky when older. The leaves of *P. suberosa* are polymorphous and are widely utilized in Sri Lankan traditional medicine to treat diabetes, hypertension, skin ailments and as a sedative (Dhawan, Dhawan & Sharma, 2004). In traditional Sri Lankan Ayurveda medicine, raw leaves of *P. suberosa* have been widely used in the management of DM. However, there is limited reported literature on hypoglycaemic activity of *P. suberosa* available supporting the ethnomedical usage of the plant. The purpose of this study was to investigate the effects of *P. suberosa* extract on glucose and lipid profile in mice. Furthermore, the study was extended to investigate the underlying mechanisms and toxic effects of the extract.

## Materials & Methods

### Chemicals

Commercial assay kits for the glucose oxidation, lipid profile, ALT and AST were purchased from Biolabo (SA, Maizy, France). All other reagents were purchased from Sigma-Aldrich Co. (St. Louis, MO, USA).

### Herbal extract and phytochemical analysis

Fresh leaves of *Passiflora suberosa* were collected from mature healthy plants from home gardens of Pelawatta area, Western Province, Sri Lanka (6.872916N, 79.888634E). The plant material was authenticated at the Royal Botanical Garden, Peradeniya, Sri Lanka and a voucher number was undertaken. Hundred grams of fresh *P. suberosa* leaves were carefully washed and shade dried. Leaves were subjected to grinding in a high speed grinder, and suspended in distilled water to obtain a final volume of 1 L. The extraction was filtered using a muslin cloth to remove debris and the yield (27.70% w/w) generated was subjected to freeze-drying at −70 °C. The obtained crude was then stored at 4 °C for future experiments. The separated aqueous leaf extracts (ALE) were dissolved in distilled water to prepare required doses.

### Phytochemical screening

The aqueous extract of *P. suberosa* leaves were subject to preliminary phytochemical screening to detect the presence of phytochemicals such as alkaloids, sterols, triterpenes, saponins, flavonoids, proanthocyanidins and anthraquinones according to previously published methods (Harborne, 1998).

### Confirmatory test for flavonoids

Aqueous extract was subjected to thin layer chromatographic studies on precoated Kieselgel 60 F_254_ using ethyl acetate: formic acid: dichloromethane: methanol (6.8: 0.2: 2.8: 0.2) as the solvent systems. Natural product reagent (NPR) was used as the visualizing agent. The developed spots were observed under UV light before and after spraying the reagent at 254 nm and 365 nm respectively.

### Test for saponins

The powdered leaves of *P. suberosa* (5 g) was placed in a test tube and 10 mL of distilled water was added and shaken vigorously for 30 s. It was then kept for 30 min and observed the froth formation.

### Experimental design

Male ICR (imprinting control region) mice weighing between 30–40 g were obtained from the Medical Research Institute, Colombo and housed in animal house (temperature: 24–25 °C, photoperiod: 12 h day and 12 h dark and relative humidity: 55–60%) at department of Biochemistry, Faculty of Medicine with free access to water. The animal handling was in accordance with the institutional guidelines (ethical clearance no. 25/14) for the care and use of laboratory animals.

### Determination of carbohydrate content

To determine the ash content, 10 g of *P. subserosa* was weighed using a silica crucible. The crucible was heated at 600 °C for 3–5 h in muffle furnace until plant material is completely charred. Subsequently, the material was cooled in a desiccator. To ensure completion of ashing process it was heated again in the furnace for further 30 min. The procedure was repeated until a constant ash weight was obtained (ash became white or greyish white). Weight of ash gave the ash content. Similarly, moisture, fat and protein contents were determined as described by Indrayan et al. (2005).

% carbohydrate = 100 –  (% of ash + % of moisture + % of fat + % of protein).

### Acute treatments

#### Fasting blood glucose concentrations

The following experimental procedure was adopted to determine the fasting blood glucose levels (BGL) of animals kept fasting overnight with free access to water. Each group consisted of 9 animals.

Group 1 –Control (1 mL of distilled water)

Group 2 –1 mL of 25 mg/kg of ALE

Group 3 –1 mL of 50 mg/kg of ALE

Group 4 –1 mL of 100 mg/kg of ALE

Group 5 –1 mL of 200 mg/kg of ALE

Following treatment, under light ether anaesthesia, 5 blood drops were collected from the tail tip of each mouse using aseptic precautions at 1 h pre-treatment and at 1, 3 and 5 h post-treatment. The serum was obtained and serum BGL was determined immediately according to the method described in the instructions manual of the standard glucose oxidase assay kit and spectrophotometer (Labomed, Inc., Los Angeles, USA).

### Random blood glucose concentrations

Eighteen mice provided free access to food and water were treated orally either with 1 mL of 50 mg/kg ALE or distilled water. Under light anaesthesia five drops of blood were collected from the tail tips 1 h prior to the treatment and at 1, 3, 5 h of post-treatment. Serum was separated and random BGLs were determined using glucose oxidase assay kit and spectrophotometer at 500 nm wavelength.

The maximum reduction in BGL was evident in animals gavaged with 50 mg/kg and further this dose is correspondent to the prescription recommended by traditional medical doctors in Sri Lanka. Hence, this dose was subjected to further studies. Eighteen mice (*n* = 9) were randomly separated in to two groups, and treated either with 1 mL of 50 mg/kg of the extract or 1 mL of ddH20 (double distilled water) for 30 successive days

### Oral glucose and sucrose tolerance tests

To analyse glucose and sucrose overloading, the method described by Ratnasooriya, Jayakody & Hettiarachchi (2004) was modified. Thirty minutes after oral treatment with 50 mg/kg ALE or distilled water, mice were loaded with 4 g/kg glucose solution (Okine et al., 2005). Consequently, after 1, 3 and 5 h of the treatment, blood samples were collected from the tails of mice and serum BGLs were determined using glucose oxidation kit at 500 nm wavelength. To evaluate sucrose tolerance, the same procedure was followed; however, instead of glucose, the mice were loaded with 5 g/kg solution of sucrose (Fujita & Yamagami, 2001).

### Intestinal glucose absorption

The glucose movement across the intestinal epithelium was determined in an isolated section of the small intestine (Dharmasiri, 2001). Briefly 18 mice were fasted overnight with free access to water. Animals were treated with 1 mL of distilled water or 50 mg/kg ALE (*n* = 9). Thirty minutes later, mice were loaded with 10 mL/kg of 50% glucose solution. At 2 h post-treatment, mice were sacrificed and their small intestines were separated and 5 mL of distilled water was infused from one end and content was collected at the other end. Content was centrifuged at 3,000 rpm for 5 min and supernatant was removed. Glucose concentration in the supernatant was estimated using glucose oxidase kit at 500 nm.

### Long term treatment

Animals were orally treated either with distilled water or 50 mg/kg of ALE for continuous 30 days. Animals were observed daily for any adverse behaviours, toxic symptoms or deaths.

#### Fasting blood glucose concentration

Since, only the fasting BGL produced significant effects with the acute treatment and traditional doctors advise to partake the herbal formulations in an empty stomach, only fasting BGLs were evaluated following chronic treatment. To investigate the long-term treatment effects of *P. suberosa* on fasting BGLs, modified methods described by Fernandopulle, Karunanayake & Ratnasooriya (1994) were adopted. After treating the mice for 30 days with the effective dose (50 mg/kg ALE) and distilled water, on day 31, the serum BGLs was evaluated immediately using glucose oxidase assay kit and spectrophotometer (Labomed, Inc., Los Angeles, CA, USA).

### Diaphragm glucose uptake

Upon autopsy, glucose uptake of isolated diaphragms was assessed after immersing them in 1 g/L glucose solution and glucose concentration was measured prior and 30 min after incubation (Subban et al., 2008). Briefly, subsequent to chronic treatment, on day 31, animals were scarified and diaphragms were removed and washed immediately with krebs-ringer solution and were immersed in 2% bovine serum albumin supplemented with tyrode ringer solution buffer containing 1 g/L glucose solution and incubated in a CO_2_ incubator (Sanyo Electric Co. Ltd., Tokyo, Japan) at 37 °C, 95% air for 30 min. Following incubation, each diaphragm was removed from incubation media and 10 µL of the media was removed to assess the glucose concentration using glucose oxidation kit and a spectrophotometer at 500 nm.

### Glycogen content in the liver and skeletal muscles

Subsequent to chronic treatment animals were scarified on day 31. Upon autopsy of animals, sections of livers and skeletal muscles were obtained and glycogen content were analysed (Seifter et al., 1950). Briefly, 100 mg of each organ was digested with boiling KOH. Following cooling down, .95% ethanol was added and heated until bubbles were formed. Tubes were cooled and centrifuged at 1,000 rpm and supernatant was discarded to obtain the residues. These were washed with 5 mL of distilled water and after several washing, anthrone reagent was added and kept in ice. Tubes were incubated at 100 °C for colour development and absorbance was measured at 620 nm. Distilled water and glucose solutions were used respectively as the blank and the standard.

### Pancreatic islet isolation procedure and beta cell mass assessment by cell staining

Animals were treated for 30 consecutive days either with 50 mg/kg or distilled water. On day 31, upon autopsy the pancreas of mice was excised, washed, and blotted and was fixed in Bouin’s fixative. After the routine processing of tissues, 4-µm thick sections were made via microtomy and stained with haematoxylin and eosin for microscopic examination (Gupta et al., 2012a; Gupta et al., 2012b).

### General toxicology and biochemical parameters

Throughout the chronic treatment phase, animals were observed daily for any toxic effect for first 4 h after the treatment and signs of overt toxicity (diarrhoea, salivation, convulsion, tremor, changes in skin and eye colour, behavioural changes, respiratory changes), motility and other parameters such as food intake, water intake, and body weight were also determined (Rangika, Dayananda & Peiris, 2015).

On day 31, animals were sacrificed by an aesthesia (chloroform) after an overnight fasting. The blood samples were obtained via heart puncture to determine the serum total cholesterol (TC), triglycerides (TAG), high-density lipoprotein cholesterol (HDL-C), and low-density lipoprotein cholesterol (LDL-C), alanine aminotransferase (ALT) and aspartate aminotransferase (AST) levels using commercial kits (Rangika, Dayananda & Peiris, 2015). The liver, spleen, heart, testes, and kidneys were removed and blotted dry to determine their wet weights. For histopathological analysis, sections of the liver were fixed in Bouin’s fixative and 4-µm thick paraffin sections were made and stained for microscopic examination.

### Statistical analysis

The results were expressed as mean ± standard error of the mean and statistically analysed by one-way analysis of variance followed by Kuskal–Wallis and Mann–Whitney *U*-test. The dose dependent was analysed using regression analysis. The level of significant was set at *p* ≤ 0.05.

## Results

### Qualitative phytochemical screening of *P. suberosa* leaf extracts

Ground leaves of *P. suberosa* were extracted using water under reflux conditions. The crude extracts thus obtained were subjected to qualitative phytochemical screening studies. The extract revealed the presence of alkaloids, unsaturated sterols, triterpenes, saponins, flavonoids, tannins and proanthocyanidins (Table 1).

**Table 1:** Qualitative phytochemical analysis of aqueous extracts of P. suberosa leaves. + Presence of constituent; − Absence of constituent.

Class of compounds	*P. suberosa* aqueous leaf extract
Alkaloids	+
Sterols	+
Triterpenes	+
Saponins	+
Flavonoids	+
Proanthocyanidins	+
Anthraquinones	–
Tannins	+

Percentage ash, moisture, fat and protein contents obtained for *P. suberosa* leaves were respectively 5.89%, 65.08%, 1.51% and 14.566%. Hence the carbohydrate content of *P. suberosa* was found to be 12.97%.

**Figure 1:**
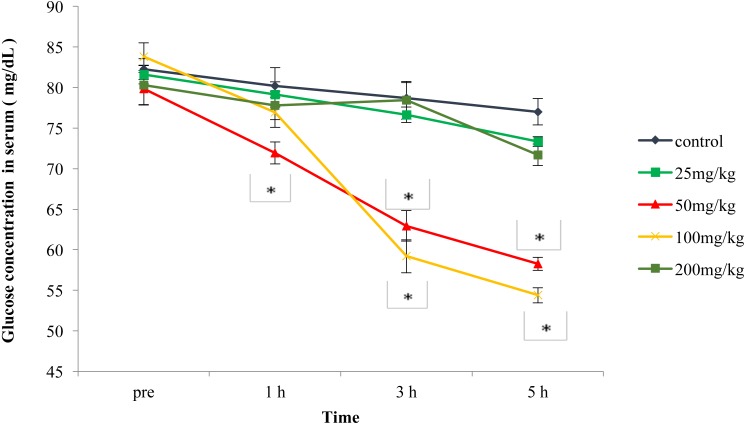
Effects of the aqueous leaf extract of *P. suberosa* (25, 50, 100 and 200 mg/kg) or control (distilled water) on fasting blood glucose levels (mg/dL). Glucose concentration (mg/dL) was measured prior to treatment and at 1 h, 3 h and 5 h post-treatment. Results are expressed as the means ± SEM, *n* = 9; ∗*p* < 0.01. File S1.

The results presented in Fig. 1 show that the fasting BGLs of 50 mg/kg and 100 mg/kg of ALE was significantly (*p* < 0.01) reduced than that of the control mice. The glucose lowering ability was markedly higher (10%, 20% and 24%, respectively at 1 h, 3 h and 5 h) in mice treated with 50 mg/kg dose than that of the 100 mg/kg (24% and 29%, respectively at 3 h and 5 h). Fasting BGL of the lowest dose (25 mg/kg) and the highest dose (200 mg/kg) was not significantly (*p* > 0.05) different from that of the control mice. The linear regression analysis revealed that the reduction of BGLs was not dose-dependent (*R*^2=^0.272).

However, the effective dose (50 mg/kg) of the ALE was unable to produce a significant (*p* > 0.05) effect on random BGLs in non-fasted mice (Table 2).

**Table 2:** Effect of *P. suberosa* on random fasting blood glucose levels in mice. Values are expressed as the means ± SEM (*n* = 9). The control groups received ddH_2_O, and the treatment group received 50 mg/kg of the extract. A random glucose level was measured prior to treatment and at 1 h, 3 h and 5 h post-treatment in the non-fasted mice. No significant difference was observed between the groups (ANOVA; Kuskal–Wallis tests).

Treatment	Pre-treatment		Post-treatment	
		1 h	3 h	5 h
Glucose concentration (mg/dL)				
Control	121.56 ± 1.47	122.49 ± 1.01	125.85 ± 2.25	126.45 ± 2.24
*P. suberosa*	124.32 ± 0.97	127.72 ± 0.61	130.67 ± 0.45	132.43 ± 0.36

Glucose tolerance of the 50 mg/kg was not significantly (*p* > 0.05) different from that of the control group. In contrast, the mice of the 50 mg/kg groups were able to produce a significant (*p* < 0.05) improve of the sucrose tolerance at 3 h and 5 h (13% and 18%, respectively) post-treatment when compared with the control group (Table 3).

**Table 3:** Effect of the aqueous extract of *P. suberosa* on oral glucose and sucrose tolerance tests in mice. Values are expressed as the means ± SEM (*n* = 9). Control groups received ddH_2_O, and the treatment groups received 50 mg/kg of the extract. The data were analysed using the parametric method, ANOVA followed by Mann Whitney *U* test.

Treatment	Pre-treatment		Post-treatment	
		1 h	3 h	5 h
Glucose Tolerance Test (Serum glucose concentration (mg/dL))				
Control	90.82 ± 1.33	148.77 ± 1.50	132.39 ± 1.32	96.07 ± 1.00
*P. suberosa*	91.06 ± 1.58	143.38 ± 1.72	121.02 ± 1.31	94.58 ± 0.73
Sucrose Tolerance Test (Serum glucose concentration (mg/dL))				
Control	97.19 ± 0.89	126.71 ± 2.16	115.62 ± 1.44	104.46 ± 1.58
*P. suberosa*	98.68 ± 0.42	130.92 ± 0.78	101.03 ± 1.80^*^	85.19 ± 0.43^*^

**Notes.**

* Values are statistically significant at *p* < 0.05.

Chronic treatment of the 50 mg/kg produced a significant (*p* < 0.01) reduction in fasting BGLs at 1 h, 3 h and 5 h (17%, 18% and 27%, respectively) when compared with the control (Table 4).

**Table 4:** Effects of long-term treatment of *P. suberosa* on fasting blood glucose levels in mice. Values are expressed as the means ± SEM (*n* = 9). The control group received distilled water, and the treatment group received 50 mg/kg of the extract. The mice were treated for 30 days.

Treatment	Pre-treatment		Post-treatment	
		1 h	3 h	5 h
Glucose concentration (mg/dL)				
Control	84.35 ± 2.36	83.34 ± 2.49	78.27 ± 2.19	75.24 ± 3.72
ALE	86.34 ± 4.26	69.01 ± 3.08^*^	63.87 ± 2.47^*^	55.24 ± 3.85^*^

**Notes.**

* Values are statistically significant at *p* < 0.05 (data analysed using one-way ANOVA and Kuskal–Wallis test).

As shown in Table 5, the aqueous extract of *P. suberosa* significantly (*p* < 0.01) inhibited glucose absorption from the lumen of the intestine by 79%; however, the treatment did not increase the diaphragm uptake of glucose when compared to the control. The glycogen content in the liver (61%) and skeletal muscles (57%) of the treated group (50 mg/kg) was significantly (*p* < 0.01) increased than that of the control group.

**Table 5:** Effects of the aqueous leaf extract of *P. suberosa* on gastrointestinal and diaphragm glucose uptake and liver and skeletal muscle glycogen content in mice. The data are presented as the means ± SEM (*n* = 9). The control group was given distilled water, and the test group was given 50 mg/kg of the extract. To measure the diaphragm glucose uptake and glycogen content in the liver and skeletal muscles, the mice were treated for 30 days. For the measurement of intestinal glucose absorption, the mice were treated with acute doses.

Parameters	Control	Treatment
Gastrointestinal absorption of glucose (mg/dL)	17.59 ± 1.54	84.5 ± 2..65^**^
Diaphragm uptake of glucose (mg/dL)	33.51 ± 0.28	32.83 ± 0.43
Glycogen content in the liver (mg/dL)	1.421 ± 0.06	3.822 ± 0.14^**^
Glycogen content in skeletal muscle (mg/dL)	3.018 ± 0.04	6.868 ± 0.16^**^

**Notes.**

* Values are statistically significant at *p* < 0.05.

** Values are statistically significant at *P* < 0.01.

The TC (total cholesterol) and the TG (triglyceradyhyde) of the 50 mg/kg group was respectively 17% and 12% lower than that of the control group indicating a significant (*p* < 0.05) cholesterol reduction. Although the HDL-C was comparable to that of the control group, it was increased by 7%. The extract did not alter both ALT and AST levels (Table 6). The mice treated chronically with 50 mg/kg ALE did not exhibit any toxic symptoms, stress and aversive behaviour. Additionally, no deaths were observed. There were no marked alterations in organ weights (Table 7), body weight, water or food intake during the treatment period (Table 7). Liver histophathology revealed no obvious effects when compared to the control.

**Table 6:** Effects of the aqueous leaf extract of *P. suberosa* on lipid profile parameters, ALT, AST levels, and organ weights of mice after chronic treatment. The data are shown as the means ± SEM (*n* = 9). The control group was given distilled water, and the treated group was given 50 mg/kg of the aqueous leaf extract.

Parameters	Control	Treatment
Lipid profile parameters		
Total Cholesterol (mg/dL)	194.67 ± 0.82	161.11 ± 2.49^**^
Triglycerides (mg/dL)	74.54 ± 6.63	65.50 ± 4.41^*^
HDL-CH (mg/dL)	34.84 ± 1.14	34.12 ± 0.97
LDL-CH (mg/dL)	15.74 ± 0.65	14.73 ± 0.53
ALT & AST levels		
ALT (IU/L)	21.31 ± 0.36	21.79 ± 0.23
AST (IU/L)	12.67 ± 0.24	12.77 ± 0.61
Organ weights		
Liver (g)	1.8682 ± 0.0689	1.8440 ± 0.0101
Spleen (g)	0.1430 ± 0.0547	0.1552 ± 0.0829
Kidney (g)	0.7146 ± 0.2001	0.6365 ± 0.0438
Testes (g)	0.6064 ± 0.0120	0.6076 ± 0.0106

**Notes.**

* Values are statistically significant at ^∗^*p* < 0.05.

HDL-CH High-density lipoprotein cholesterol LDL-CH Low-density lipoprotein cholesterol ALT Alanine aminotransferase levels AST Serum aspartate aminotransferase

**Table 7:** Effects of the aqueous leaf extract of *P. suberosa* on food intake, water intake and bodyweight change in mice after chronic treatment. The data are shown as the means ± SEM (*n* = 9). No statistically significant differences were observed between values. The control group was given distilled water, and the treated group was given 50 mg/kg of the extract. Data were analysed using one-way ANOVA and Kuskal–Wallis tests.

Parameters	Treatment	# of weeks			
		1	2	3	4
Food intake (g)	Control	25.01 ± 0.73	26.05 ± 0.66	28.06 ± 0.92	27.61 ± 0.64
	50 mg/kg of ALE	24.62 ± 0.62	25.71 ± 0.72	29.11 ± 0.82	28.66 ± 0.74
Water intake (mL)	Control	40.17 ± 0.83	38.62 ± 0.59	42.58 ± 0.45	38.94 ± 0.74
	50 mg/kg of ALE	42.65 ± 0.83	40.81 ± 0.93	40.83 ± 0.30	39.33 ± 0.83
Body weight (g)	Control	36.09 ± 0.70	37.82 ± 0.72	39.56 ± 0.82	41.20 ± 0.80
	50 mg/kg of ALE	35.17 ± 0.80	36.95 ± 0.87	38.70 ± 0.93	40.33 ± 0.88

Tissue sections obtained from the pancreas of both the control group and the group receiving 50 mg/kg of ALE of *P. suberosa* did not show any difference in the size of the islets of Langerhans between the control and treated animals (File S1).

## Discussion

Here for the firs time we report hypoglycaemic activity and few mechanisms of actions of the aqueous extract of *P. suberosa*. Following 1, 3 and 5 h of treatment with 50 mg/kg dose, the ALE lowered the fasting BGLs. Similarly, 100 mg/kg dose showed reduction in fasting BGL at 3 and 5 h post-treatment. A significant lowering of fasting blood glucose concentrations were observed with long-term treatment of *P. suberosa*. Typically, in traditional medicinal practice, patients are recommended to consume natural remedies early in the morning (Rangika, Dayananda & Peiris, 2015), and the present results assist to justify this practice.

The current results revealed that the hypoglycaemic effect produced by the extracts was not dose-dependent. Particularly, the hypoglycaemic effect produced by the highest dose was lower than that of 100 and 50 mg/kg doses. Typically, the 50 mg/kg dose is prescribed by Sri Lankan traditional doctors and hence, this dose was subjected to further analyses. However, all the doses produced a decrease in BGL during the preliminary analysis. The blood glucose reduction is most likely a cumulative interaction between phytochemical components of the extract. It is possible that higher doses may exhibit dual action of activation and inhibition of blood glucose reducing mechanisms. Previous literature (Shewamene, Abdelwuhab & Birhanu, 2015) reported that presence of antagonistic molecules plant extracts could diminish the activity of the plant extract (Alarcon-aguilar et al., 2000). Hence, in low dose, the concentration of antagonistic compounds may be lower, thus allowing the extract to produce the observed hypoglycaemic effect (Singh et al., 2007). However, isolation of active compounds and further analysis are required for definite conclusions.

The current results exhibited a pattern of decrement activity of blood glucose with increasing time even up to 5 h of post-treatment. Such delayed effects may be due to active components in the extract that require a lag phase to reach sufficient concentrations at target sites. Similar delayed hypoglycaemic pattern was reported with plants such as *Pylanthus debilis* L (Wanniarachchi, Peiris & Ratnasooriya, 2009), and *Nycanthus arbor-tristis* L (Rangika, Dayananda & Peiris, 2015).

Starvation could result in reduced blood glucose levels due to oxidation of glucose. However, according to the present results, the food intake in the treated group remained unchanged and was comparable to the control group. Hence, the observed decreased blood glucose levels may not be due to impaired carbohydrate intake (Uebanso et al., 2007). *P. suberosa* did not exhibit a significant effect in decreasing the blood glucose concentration after an oral glucose challenge, suggesting that the active hypoglycaemic mechanisms do not implicate pancreatic mechanisms such as stimulating insulin release from the pancreatic β-cells or insulin mimicry action (Gupta et al., 2012a; Gupta et al., 2012b). Histopathological studies revealed unaltered pancreatic β-cells, which confirms that the extract did not stimulate the insulin release from the pancreatic cells. However, the substantial improvement in oral sucrose tolerance at 3 and 5 h indicating that the glucose decreasing activity exhibited by *P. suberosa* might be due to an alpha-glycosidase inhibitory mechanism involved in maintaining the blood glucose levels of mice. Alpha-glycosidase hydrolyses complex carbohydrates to produce monosaccharide glucose, which is absorbed through the small intestine into the hepatic portal vein; therefore, its inhibition can significantly inhibit the increase in blood glucose (Smith, Marks & Lieberman, 2005). Thus, inhibitors of alpha-glycosidase have the ability to reduce glucose levels through delaying the digestion and subsequent absorption of carbohydrates. Phytochemical screening of *P. suberosa* leaves revealed the presence of flavonoids and tannins. According to Brahmachari (2011), flavonoids exhibit glycaemic control and also could regulate the rate-determining enzymes vital for metabolic pathways of carbohydrate. Consumption of added sugars or sucrose in humans has increased over recent decades, and a drug capable of inhibiting alpha glycosidase activity similar to *P. suberosa* has a high potential to become a useful antidiabetic drug (Malik, Schulze & Hu, 2006). However, further investigations are required to clarify the alpha glucosidase inhibition activity.

Previous literature indicates the importance of plant fibre or compound carbohydrates, including soluble fibres, to manage diabetes via inhibition of glucose absorption form the intestinal epithelium (Lerman-Garber et al., 1994; Gray & Flatt, 1997). The extract of *P. suberosa* exhibited 12% of carbohydrates and most of dietary fibre present in plant leaves are insoluble fibres (Marlett & Cheung, 1997). Hence, the retardation of glucose absorption across the intestinal epithelium could be attributed to presence of physical barriers, such as complex fibre particles, that can entangle the glucose molecules in the intestine inside the network of fibre particles to prevent the rise of blood glucose levels (Ahmed, Sairam & Urooj, 2011). Clumping of fibre particles could result in forming a gelatinous matrix, resulting in delaying of the gastric emptying and impairment of glucose absorption (Gray & Flatt, 1997). The plant extracts could inhibit glucose transport across the intestinal membrane by a non-competitive type of inhibition of the intestinal glucose transporter proteins, GLUT1 and SGLT1 (Therasa, Thirumala & David, 2014). Thus, the observed inhibition of glucose absorption through the intestinal lumen may either be due to the presence of high fibre content in the extract or the inhibition of co-transporters or both.

Administration of the plant extract for 30 consecutive days exhibited a reduction in serum BGLs in mice kept deprived of food overnight. This reduction in BGL occurs in conjunction with the accumulation of glycogen in the liver and in skeletal muscles. Glycogen is the primary storage form of glucose in various tissues, especially in skeletal muscles and liver. Liver is the vital organ that maintains glucose uptake and storage via glycogenesis and regulates the release of glucose by glycogenolysis and gluconeogenesis (Anderson et al., 2015). Escalation in glycogen content in the liver denotes an increased release of insulin. Increased glycogen content in the skeletal muscle indicates enhanced cellular transport of glucose (Das et al., 2014). Insulin hormone is a key regulator of synthesis of glycogen in both skeletal muscles and hepatic tissues. Insulin activates the glycogen synthase enzyme to promote the synthesis of glycogen and thus stimulating the glucose uptake. Due to insulin deficiencies occur in diabetic conditions, a decrease of glycogen reserve in the skeletal muscles and liver tissues can be observed in diabetic patients (Eliza et al., 2009). A significant increase of glycogen content by the extract of *P. suberosa* indicates the reactivation of the glycogen synthase system (Parveen, Mujeeb & Siddiqui, 2010), thus indicating that the plant extract exerts a direct influence on muscle glycogen metabolism and glucose uptake and hence the plant extract could be highly beneficial to prevent loss of muscle physique in diabetic conditions.

Glycaemia is associated with hyperlipidaemia. Alteration in the serum lipid profile is evident in diabetes resulting in an increased risk for coronary heart diseases (Krentz, 2003). Lipid peroxidation has been established as one of the characteristic features of chronic diabetes. Diabetic conditions cause the breakdown and increased mobilization of free fatty acids from peripheral deposits. Additionally, lipolysis by hormones results in elevated lipid levels, which increases the risk of myocardial dysfunction (Rojop et al., 2012). The extract prevented the elevation of serum TCH and TG levels in mice indicating a cardio-protective potential of the extract. Presence of insoluble fibres could result in improvement of lipid profile (Marlett & Cheung, 1997). Inhibition of lipolysis could lead to reduction of TG (Ben Khedher et al., 2018). Similarly, although not significantly, the extract averted the increase in LDL-CH, further decreasing the risks of arteriosclerosis. The consequent reduction in BGL observed in the present study may lead to the control of hormones and inhibition of lipolysis involving plasma lipoprotein lipase, thus affecting triacylglyceride catabolism (Xie et al., 2007).

Diabetes plays a key role in liver damages via up regulating hepatic superoxide dismutase and reducing hepatic malondialdehyde (Hickman & Macdonald, 2007). The serum activities of ALT, AST, and ALP are biomarkers of hepatic injury (Ayepola et al., 2013). ALT and AST are transaminase enzymes that catalyse amino transfer reactions are vital in amino acid catabolism and biosynthesis. In current study, the levels of ALT and AST remained unaffected indicating that the extract did not induce any palpable liver damages. This observation was confirmed by the liver weights and histological evaluations, which did not indicate any obvious abnormalities. Furthermore, the mice treated with 50 mg/kg of ALE did not exhibit any overt signs of clinical toxicity, behavioural changes. The body weights and water and food intake remained unaltered throughout the study, which confirmed that the ALE of *P. suberosa* leaves could be used effectively to manage diabetes.

## Conclusions

As synopses of the present results, the ALE showed that the herbal preparations are capable of exercising glycaemic regulation via inhibiting the glucose absorption by the intestinal epithelium, enhancing the synthesis of glycogen in the liver and skeletal muscle, and by improving the glycogen storage in the peripheral tissues. Furthermore, the extract showed a capacity to improve the lipid profile. However, it is worthwhile to evaluate sedative potential of the leaf extract and further isolation of active compounds are also warranted.

##  Supplemental Information

10.7717/peerj.4389/supp-1 File S1 Data for acute treatment of P. subersoa (25, 50, 100 and 200 mg/kg) or control (ddH2O) on fasting blood glucose levels (mg/dL) 25, 50, 100 and 200 mg/kg of the extract or control (ddH2O) on fasting blood glucose levels (mg/dL). Data are given for 9 animals.

10.7717/peerj.4389/supp-2 File S2 Given data for fasting blood sugar after acute and long-term treatments, oral glucose and sucrose tolerance, glucose absorption by the intestine, glucose uptake by the diaphragm, glycogen content in the liver and skeletal muscles, lipid profile and kidney Data are given for 9 animals.

10.7717/peerj.4389/supp-3 File S3 Schematic diagram of methodology Methodology of determination of fasting blood glucose levels, glucose & sucrose tolerance, absorption of glucose from the small intestine, glycogen content in the liver and skeletal muscles, diaphragm uptake of glucose, pancreatic beta cell visualization are included.

10.7717/peerj.4389/supp-4 Supplemental Information 1 Pancreatic beta cell mass of control mice Pancreatic beta cells of mice given distilled water after 30 days.

10.7717/peerj.4389/supp-5 Supplemental Information 2 Beta cell mass of mice treated with aqueous leaf extract Cells stained with hemotoxylin and eosin after chronic treatment. The arrow indicates islets of Langerhans. Mag × 400.

## Additional Information and Declarations

### Data Availability

The following information was supplied regarding data availability: The data is provided as a Supplemental File.
