# Supplementary material for: Hypoglycemic and hypolipidemic activity of aqueous leaf extract of Passiflora suberosa L
Source: PeerJ. 2018 Feb 20;6:e4389. doi: 10.7717/peerj.4389 (PMC5824672; doi:10.7717/peerj.4389)
Supplement: File S3 — Methodology of determination of fasting blood glucose levels, glucose & sucrose tolerance, absorption of glucose from the small intestine, glycogen content in the liver and skeletal muscles, diaphragm uptake of glucose, pancreatic beta cell visualization are included. [file peerj-06-4389-s003.pptx]

## Slide 1
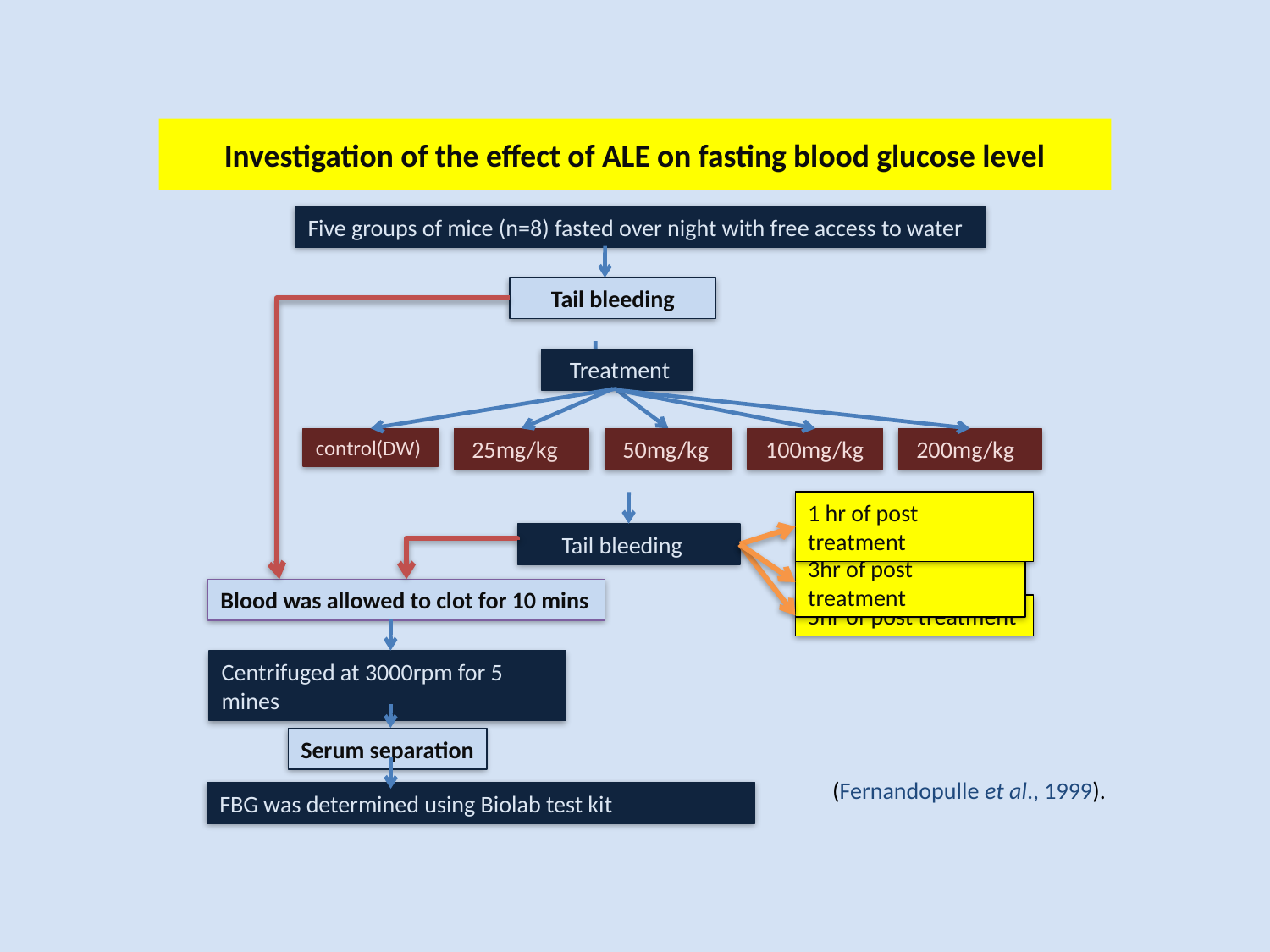

# Investigation of the effect of ALE on fasting blood glucose level
Five groups of mice (n=8) fasted over night with free access to water
Tail bleeding
 Treatment
control(DW)
 25mg/kg
 50mg/kg
 100mg/kg
 200mg/kg
1 hr of post treatment
 Tail bleeding
3hr of post treatment
Blood was allowed to clot for 10 mins
5hr of post treatment
Serum separation
FBG was determined using Biolab test kit
Centrifuged at 3000rpm for 5 mines
(Fernandopulle et al., 1999).

## Slide 2
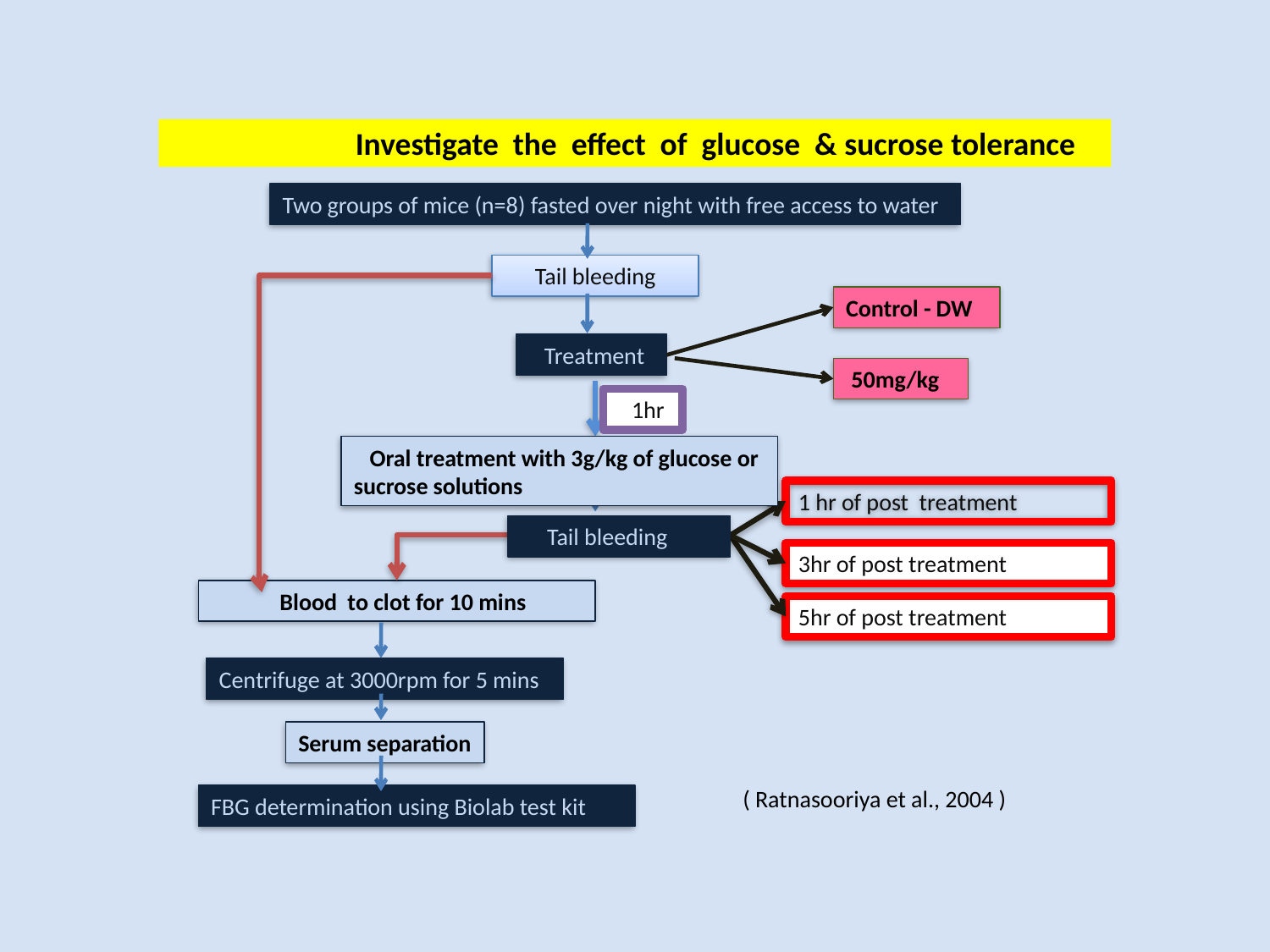

Investigate the effect of glucose & sucrose tolerance
Two groups of mice (n=8) fasted over night with free access to water
Tail bleeding
Control - DW
 Treatment
 50mg/kg
1 hr of post treatment
3hr of post treatment
 Blood to clot for 10 mins
5hr of post treatment
Serum separation
FBG determination using Biolab test kit
 1hr
 Oral treatment with 3g/kg of glucose or sucrose solutions
 Tail bleeding
Centrifuge at 3000rpm for 5 mins
( Ratnasooriya et al., 2004 )

## Slide 3
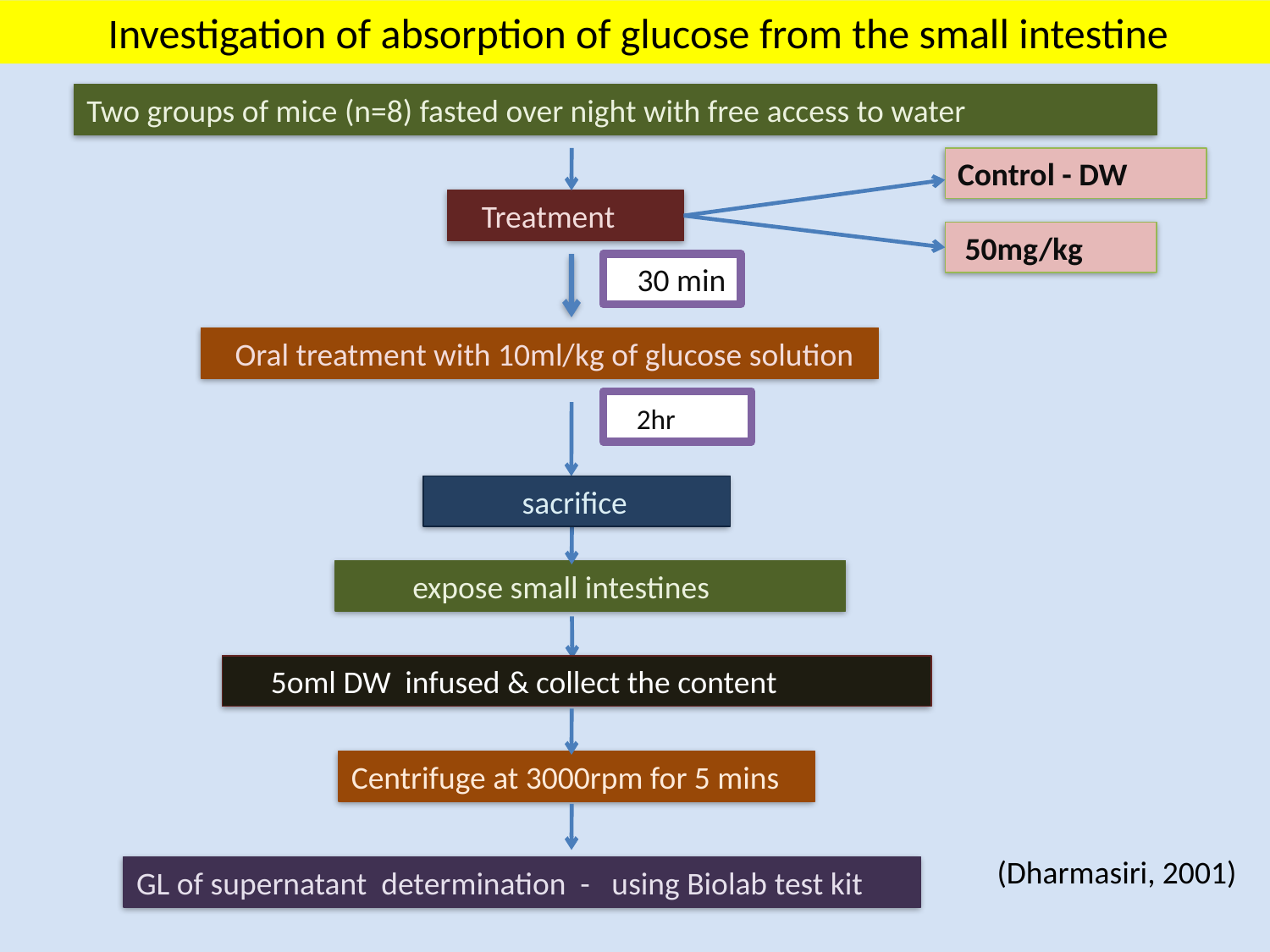

Investigation of absorption of glucose from the small intestine
Two groups of mice (n=8) fasted over night with free access to water
Control - DW
 Treatment
 50mg/kg
 expose small intestines
GL of supernatant determination - using Biolab test kit
 30 min
 Oral treatment with 10ml/kg of glucose solution
 2hr
 sacrifice
 5oml DW infused & collect the content
Centrifuge at 3000rpm for 5 mins
 (Dharmasiri, 2001)

## Slide 4
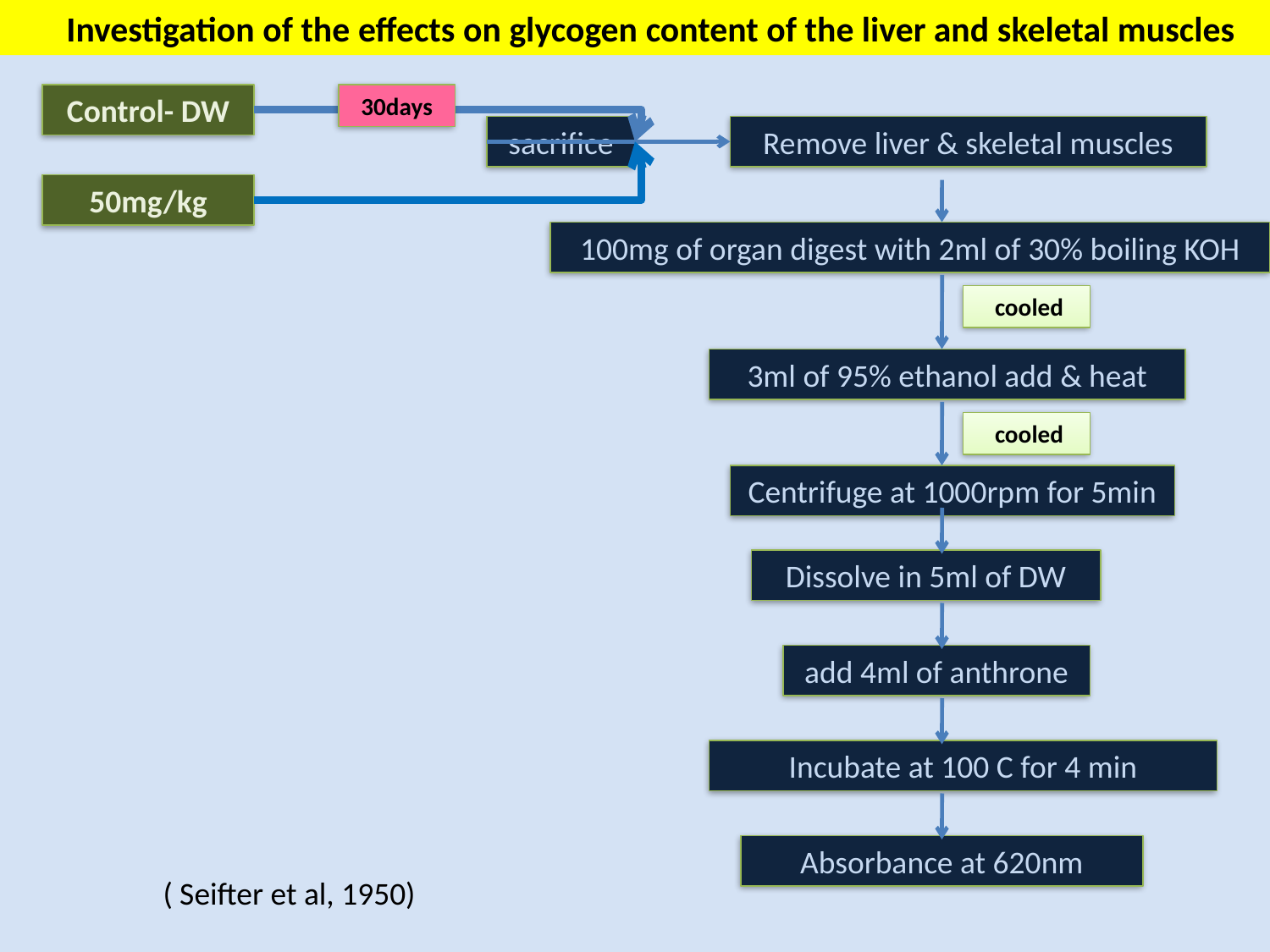

Investigation of the effects on glycogen content of the liver and skeletal muscles
Control- DW
30days
sacrifice
 Remove liver & skeletal muscles
50mg/kg
100mg of organ digest with 2ml of 30% boiling KOH
 cooled
3ml of 95% ethanol add & heat
 cooled
Centrifuge at 1000rpm for 5min
Dissolve in 5ml of DW
 add 4ml of anthrone
Incubate at 100 C for 4 min
Absorbance at 620nm
( Seifter et al, 1950)

## Slide 5
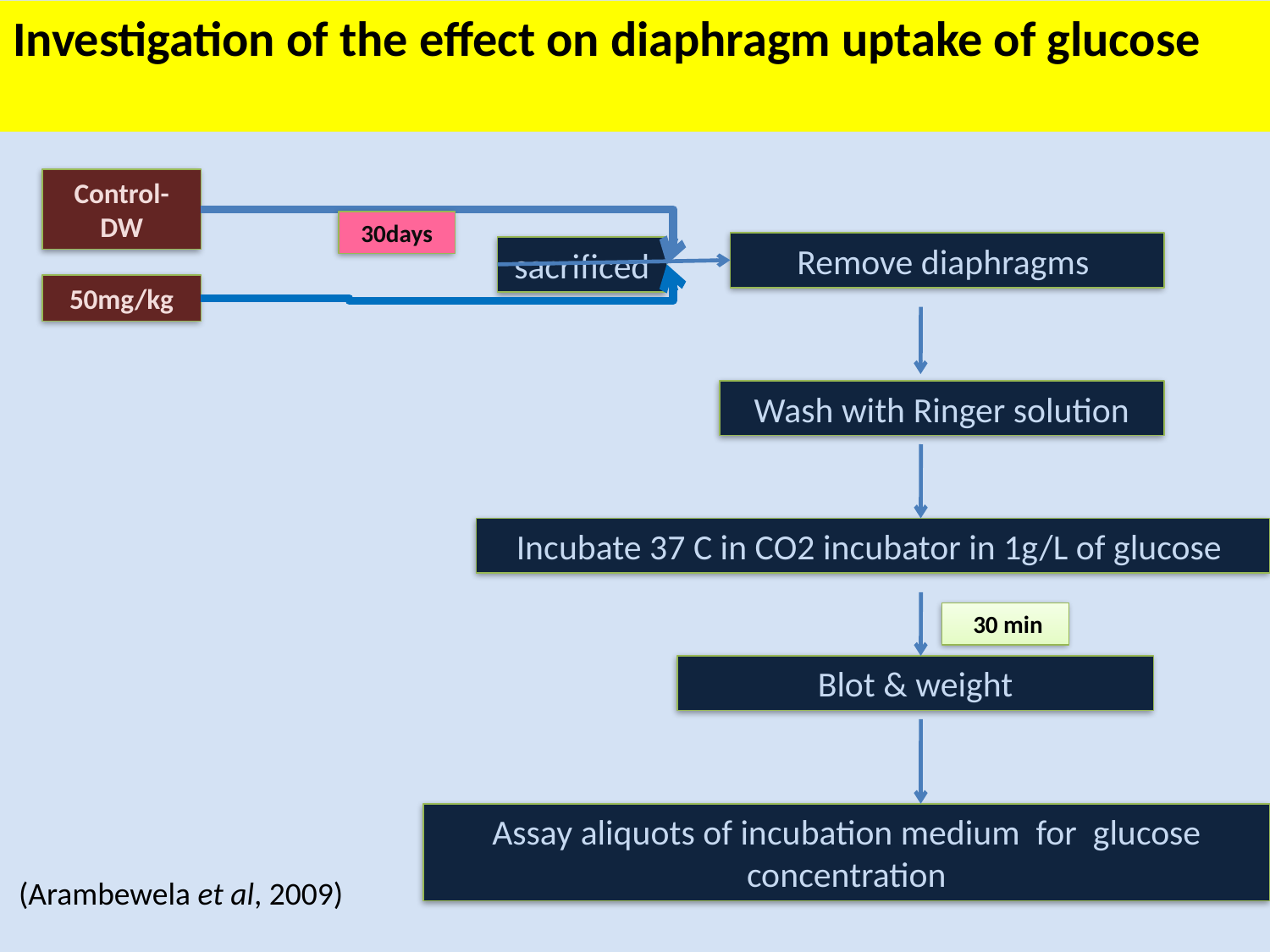

Investigation of the effect on diaphragm uptake of glucose
Control- DW
30days
Remove diaphragms
sacrificed
50mg/kg
Wash with Ringer solution
Incubate 37 C in CO2 incubator in 1g/L of glucose
 30 min
Blot & weight
Assay aliquots of incubation medium for glucose concentration
(Arambewela et al, 2009)

## Slide 6
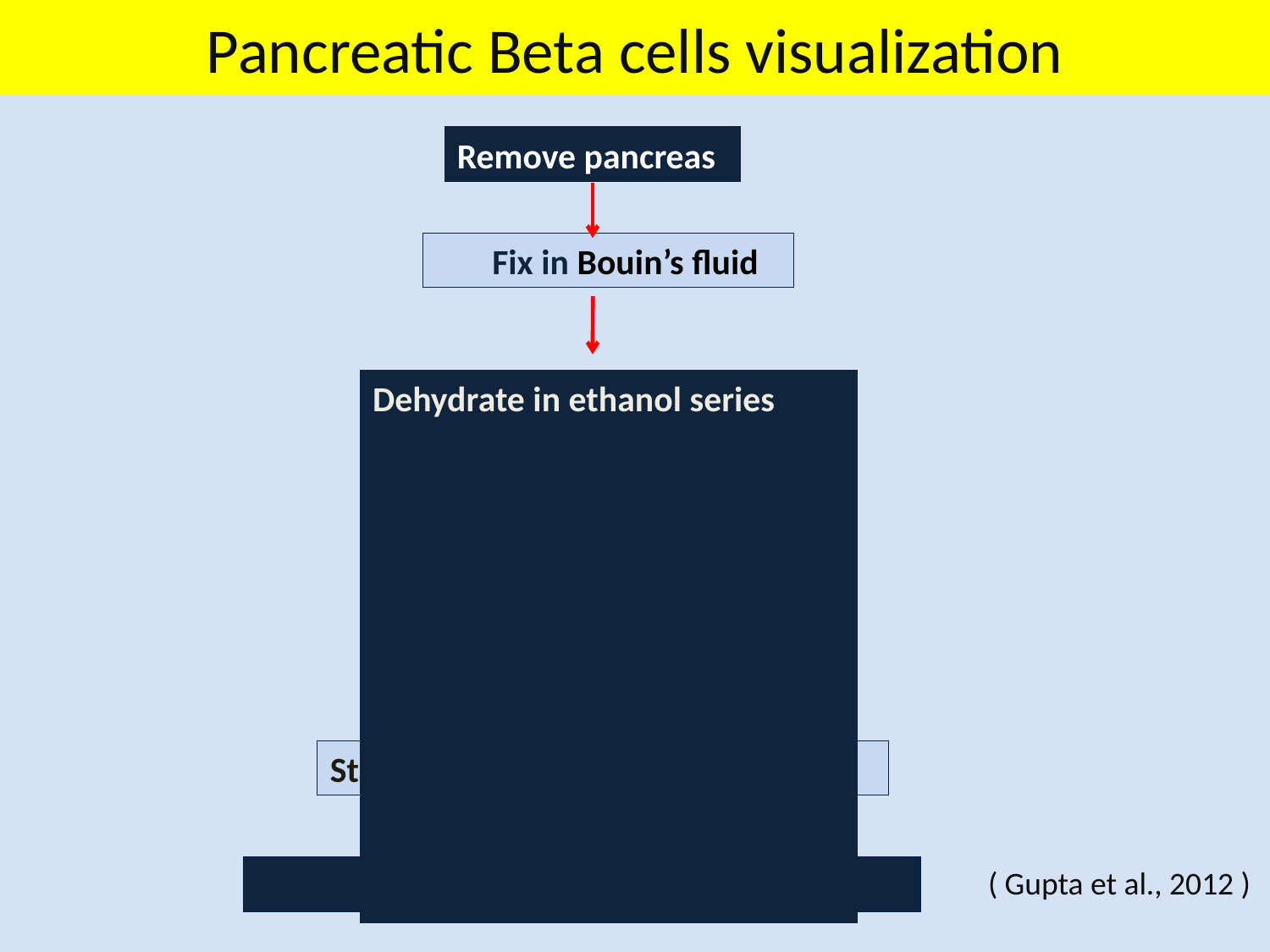

# Pancreatic Beta cells visualization
Remove pancreas
 Fix in Bouin’s fluid
Dehydrate in ethanol series
Embed in paraffin
 Obtain sections
Stain with hematoxylin and eosin
 Examine under light microscope
( Gupta et al., 2012 )
