# Supplementary figures and images for: Hypoglycemic and hypolipidemic activity of aqueous leaf extract of Passiflora suberosa L
Source: PeerJ. 2018 Feb 20;6:e4389. doi: 10.7717/peerj.4389 (PMC5824672; doi:10.7717/peerj.4389)

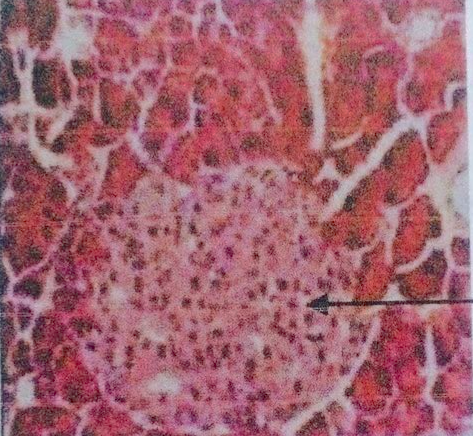

Supplement: Supplemental Information 1 — Pancreatic beta cells of mice given distilled water after 30 days. [file peerj-06-4389-s004.tiff]

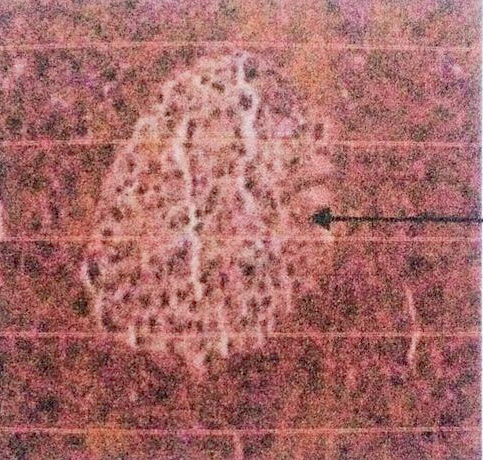

Supplement: Supplemental Information 2 — Cells stained with hemotoxylin and eosin after chronic treatment. The arrow indicates islets of Langerhans. Mag × 400. [file peerj-06-4389-s005.tiff]
